# Uric acid levels are independent of anti-*Saccharomyces cerevisiae* antibodies (ASCA) in Crohn’s disease: A reappraisal of the role of *S. cerevisiae* in this setting

**DOI:** 10.1080/21505594.2018.1496779

**Published:** 2018-08-08

**Authors:** B. Sendid, S. Jawhara, H. Sarter, P. Maboudou, C. Thierny, C. Gower-Rousseau, J. F. Colombel, D. Poulain

**Affiliations:** a Inserm, Univ. Lille, CHU Lille, UMR995-LIRIC- Lille Inflammation Research International Center, Lille, France; b CHU Lille, Parasitologie-Mycologie, Institut de Microbiologie, Lille, France; c CHU Lille, laboratoire de Biochimie, Institut de Biochimie et Biologie Moléculaire, Lille, France; d Division of Gastroenterology, Icahn School of Medicine at Mount Sinai, New York, NY, USA

**Keywords:** Anti-*Saccharomyces cerevisiae* antibodies (ASCA), Crohn’s disease, Inflammatory Bowel Diseases, pathogenesis, uric acid, yeasts

The incidence of Crohn’s disease (CD) in industrialized countries has increased steadily due to improvements in the standard of living since the Second World War[1]. CD is characterized by an uncontrolled immune response within the intestinal mucosa. The activation and/or chronicity of the inflammatory response depends on genetic susceptibility to various environmental stimuli, including the intestinal flora [2]. The development of CD is associated with the sequential appearance of antibodies against microbial molecular motifs, the levels of which remain stable once the disease has become established. These antibodies, initially used for diagnostic purposes, are now used for CD clinical management. The number of antigenic targets and the magnitude of the response, are indicative of disease severity [3], are now taken into account when defining the nature of initial treatment[4]. Similarly, their pre-existence to CD onset is increasingly considered in surveys of high risk populations[5]. Despite their widespread use, the mechanisms of generation and persistence of these antibodies remain unknown.

Anti-*Saccharomyces cerevisiae* antibodies (ASCA) are the most widely used of these antibodies. A recent paper involving murine models reported that the inability of *S. cerevisiae* to catabolize purines affects host metabolism through uric acid (UA) production and that this may negatively affect the course of inflammatory bowel disease (IBD) [6]. As an argument in favour of this hypothesis, a correlation between ASCA and UA was reported in healthy subjects. Because of the impact of this conclusion, which suggests that *S. cerevisiae* is potentially pathogenic in human beings, especially those susceptible to developing CD, we carried out experiments to investigate this correlation. We measured UA and ASCA levels in our cohorts of IBD patients and controls. Our conclusion was that this correlation does not exist and we think it important to publish these data and to extend the discussion to the significance of ASCA, the role of *S. cerevisiae* and, more generally, the role of yeasts in CD.

Our investigations involved two cohorts of IBD patients included in previous studies comprising ASCA levels determination. Sera collected from four groups of patients were analyzed. The first serum repository (CD1) consisted of 28 sera collected from 28 patients (nine male/19 female; mean age: 27.5 years; age at diagnosis: 21 years; location of disease: ileal (L1; *n *= 22), colonic (L2; *n *= 0), ileocolonic (L3; *n *= 6) selected on the basis of high ASCA levels associated with CD complications needing ileocecal resection. The sera were taken the day before surgery [Effect of Fluconazole on the Levels of Anti-*Saccharomyces cerevisiae* Antibodies (ASCA) After Surgical Resection for Crohn’s Disease. Multicenter, Randomized, and Controlled in Two Parallel Groups Versus Placebo. ClinicalTrialsgov ID: NCT02997059]. The second serum repository (CD2) came from genetic and functional studies of IBD patients [French Ministry for Health, Programme Hospitalier de Recherche Clinique, Etudes génétique et fonctionnelle des patients atteints de maladies inflammatoires chroniques de l’intestin; Grant 2006-Lille19-01]. This consisted of 85 sera from 49 CD patients (14 male/35 female; mean age: 26 years; age at diagnosis: 20 years; location of disease: L1 (*n *= 11), L2 (*n *= 4), L3 (*n *= 34). The third serum repository (UC) was collected from 36 patients with ulcerative colitis (male/female: 20/16; mean age: 34 years, age at diagnosis: 31 years) with the following phenotypes at diagnosis: proctitis (E1; *n *= 11), left colitis under splenic flexure (E2; *n *= 15) and colitis above splenic flexure (E3; *n *= 10). Finally, the fourth group (Controls) consisted in 30 sera from healthy blood donors (14 male/16 female; mean age: 37 years) obtained from Etablissement Français du Sang, Nord de France, Lille, France. ASCA levels were determined by ELISA (IBDX gASCA; Glycominds) [7] . Serum UA was measured by enzymatic colorimetric assay using a commercially available kit from Roche Diagnostics (reference ranges for serum UA in healthy adults are 25–70 mg/L).

**Figure 1.**
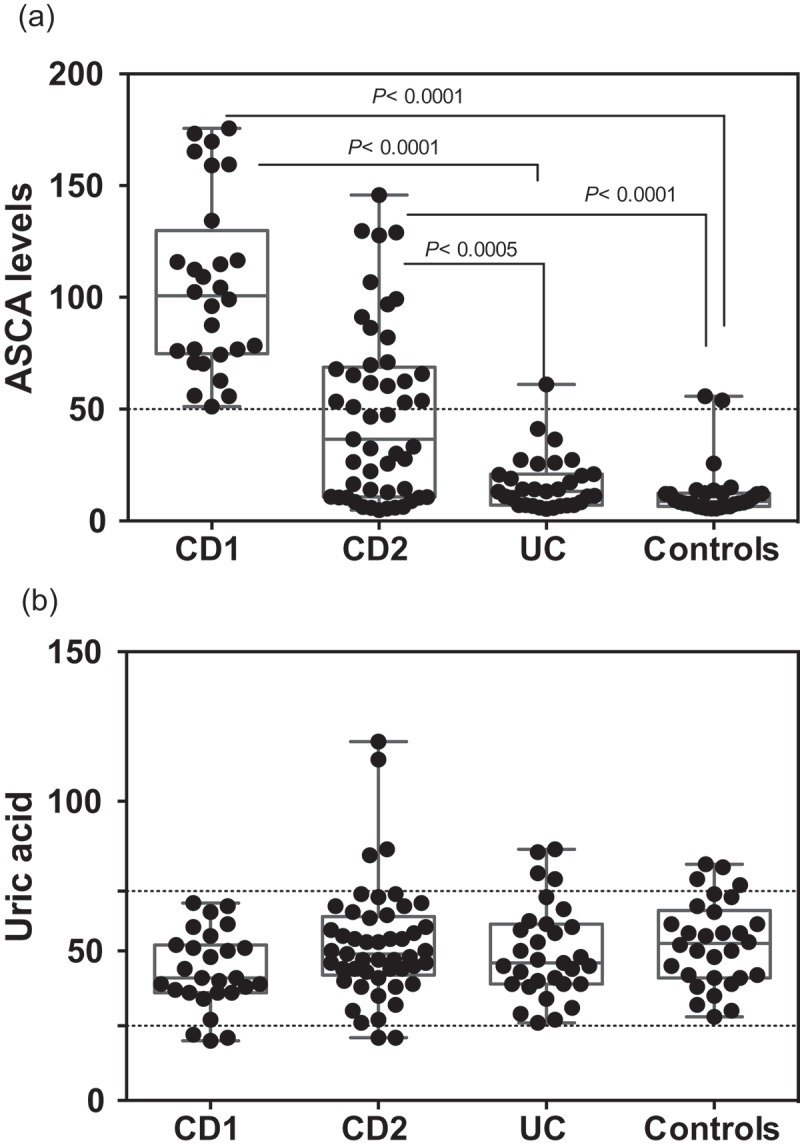
ASCA (a) and Uric acid levels (b) in the different populations examined. Dotted lines represent the limit of significance for ASCA and the reference ranges in healthy subjects for UA.

**Figure 2.**
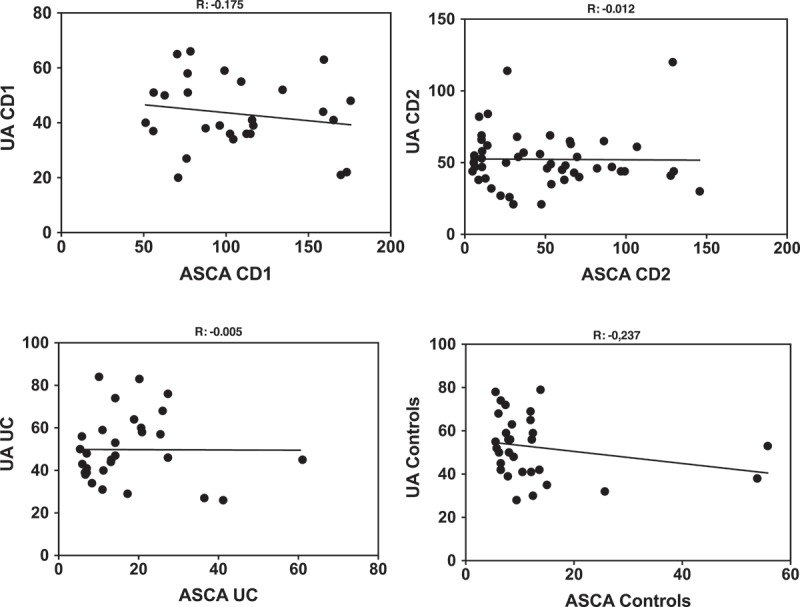
Correlations between ASCA and Uric acid levels in the CD patients groups (CD1 and CD2), Ulcerative colitis patients (UC) and Controls.

The levels of ASCA and UA determined in the different populations are shown in Figure 1. ASCA levels in the CD1 cohort were all above the limit of significance (50 AU), due to the mode of selection, with some of them exhibiting very high values typically associated with complicated disease needing surgery[3] . ASCA values in patients from the CD2 cohort were widely distributed with lower values corresponding to patients with a colonic location and/or older age at onset. Among the UC patients, only one was at the limit of significance of the test, in coherence with its initial indication for the differential diagnosis of the two forms of IBD [8]. Finally, the presence of ASCA in the non-IBD population (in the order of 6–7%) is consistent with that described previously[9]. These results, revealing very significant differences between the two IBD forms and controls, validated the choice of our cohorts. In terms of UA levels (Figure 1(b)), a striking homogeneity was observed between the IBD groups and controls. With few exceptions (i.e. not belonging to the high ASCA levels group (CD1), all subjects were within the normal range of UA values and no significant difference was found between the groups. When investigating correlations between ASCA and UA to explore the previous statement that host immune reaction to “commensal yeasts” positively correlated with UA production [6], we did not find any correlations in any of our groups (Figure 2). The correlation was close to zero in the CD2 and UC groups and even presented a trend towards negativity in the CD1 group and controls.

ASCA remain the most widely used test for the diagnosis of CD and have led to more than 400 publications on this subject. An ELISA test using mannan from *S. cerevisiae* as an antigen to detect antibodies in CD patients sera was first described by our group in 1996[10]; sequential depolymerization of the mannan molecular complex showed that the discriminating epitopes consisted of α-1,3 Man at the non-reducing end of tri or di α–1,2 mannosides [10].

In a second paper, we reported the interest of this test when combined with the detection of anti-neutrophil cytoplasmic antibodies (ANCA) for the differential diagnosis of CD vs. UC and designated the antibodies detected as ASCA [8] in order to confer homogeneity between acronyms of antibodies for differentiating the two forms of IBD. This was the first mention of the term ASCA in the literature. In retrospect, it was probably an unfortunate choice since it cast suspicion on *S. cerevisiae*whose merite resides in being able to synthesize and assemble an extremely vast repertoire of oligomannosides and oligomannosidic epitopes in mannan. In this respect, it is interesting to note that most of our current knowledge about genes controlling the early steps of glycosylation of human molecules originates from studies on mannan mutants of *S. cerevisiae* [11]. With hindsight and from an immunochemical point of view it would probably have been more rational to name these antibodies, whose epitope has been confirmed by others [12], anti-oligomannose antibodies.

In the present study, we once again confirmed what has been established over several decades about the prevalence and evolution of ASCA and associated phenotypes in CD, UC and healthy controls [3,8,13]. Interestingly, it has recently been shown that, besides CD, ASCA may also be associated with other diseases such as autoimmune diseases, including celiac disease [14] or physiological disorders[15] . Regarding UA, the levels encountered in our cohorts were well within the normal range for males and females, and no differences were found between IBD patients and controls. Regarding the correlation between ASCA and UA reported by Chiaro *et al*. [6], we were unable to confirm the correlations in any of the groups examined, and even found a trend towards a negative correlation in the control population. The reasons for these differences are unknown, but an explanation may reside in an unforeseen correlation in their control population which was unrelated to *S. cerevisiae*. High levels of UA are regularly reported in patients with metabolic disorders associated with diabetes or obesity [16] and the presence of ASCA has been reported several times in the same patients, particularly in association with obesity [17]. Thus, if the cohort studied by Chiaro *et al*. [6] included subjects with excess of weight it is possible that this could be the origin of the correlations they observed. By contrast in a recent study investigating ASCA and the presence of *S. cerevisiae* in the diet, no association was found between yeast-containing foods and ASCA IgG-positivity, or between yeast-containing foods and fat mass [15]. Thus, our study does not support the ASCA-based hypothesis derived from studies in mice that *S. cerevisiae* could worsen colitis by affecting host purine metabolism leading to increased UA production. Regarding the contrast between the conclusion of Chiaro’s study presenting *S. cerevisiae* as an important pathogen and scientific and clinical evidence gathered so far in basic and medical mycology, this lack of confirmation is not surprising. First, *S. cerevisiae* cannot be qualified as an “endosaprophyte”, which would mean that the normal condition of life for this species is the gut environment in which it is able to reproduce and disseminate[18]. This niche has been colonized by a limited number of fungal species which can survive and reproduce in the human gut environment, including *Candida albicans* and *C. glabrata*. These yeasts have been studied extensively in experimental models for their adaptation to commensalism and their status as an opportunistic pathogen in relation to iatrogenic or intrinsic disturbances of host homeostasis[19].

Genetically, despite sharing homologies with *C. albicans* and more particularly with *C. glabrata, S. cerevisiae* lacks most of the traits required to develop in the human gut and be an opportunistic pathogen [19]. Accordingly, isolation of live S. *cerevisiae* in the medical mycology laboratory is rare. Additionally, confusion has recently arisen from metagenomic analyses which regularly detect *S. cerevisiae* DNA in stools. *S. cerevisiae* is as a very common component of human food (bread, beer or food additives) and the presence of *S. cerevisiae* DNA in stools reflects its transit rather than its development inside the host. This was demonstrated by recent convincing experiments addressing the important question of discrepancies between conventional mycological methods and NGS analysis which, among other points, clarified the status of *S. cerevisiae* [20]. As stated in an excellent review, the ability of some *S. cerevisiae* strains to grow at 37°C and the opportunities for repeated introduction render it among the most commonly detected fungal species in faecal samples[20]. Thus, in this setting it likely contributes to gut microbial ecology. However, casting suspicion on the safety of an ancestral ingredient of human food worldwide and generating suspicion for billions of human beings deserves debate.

In the middle of the last century, attention was drawn to the threat of invasive fungal infections (IFIs) in the hospital environment. Due to longer periods of immunosuppression, the morbidity and mortality from IFIs continue to increase despite an impressive therapeutic arsenal. In parallel, the spectrum of fungi involved has moved from well-known opportunistic pathogens with characterized virulence factors to other species previously categorized as harmless but finding, in these debilitated patients, conditions favourable to *in vivo* growth [21]. *S. cerevisiae* is among the species which are characterized as “Generally Recognized as Safe (GRAS)” microorganisms. Numerous studies have revisited the virulence status of *S. cerevisiae* strains distributed in clinical isolates, industrial strains (wine, beer, bakery) and probiotic strains (var. *boulardii*). Such studies have included *in vitro* analysis of physiological traits (i.e. the ability to grow at body temperature, pseudo-hyphal growth, protease and phospholipase secretion) [22] and *in vivo* analysis in experimental models of infection/colonization [23,24]. These studies demonstrated that there are obvious differences in pathogenic potential of strains although the reasons for this are not yet understood. A comprehensive review concluded that due to high genotypic and phenotypic heterogeneity a polyphasic approach combining genetic, *in vitro* and *in vivo* exposure studies is needed to characterize the pathogenic potential of *S. cerevisiae* strains [25]. Furthermore, host dependence is another trait revealed in convincing *in vivo* experiments showing differences in responses depending on the *S. cerevisiae* strain and origin of macrophages [26]. To complete this very complex picture, it has been shown that, like *C. albicans* [27], microevolution of probiotic or food strains occurs in human hosts which does not necessarily impose a selective pressure toward higher virulence/pathogenicity [26]. Since all *S. cerevisiae* strains present in human environments are obviously not identical, strict characterization of the strains involved in experiments is required. Unfortunately, the single *S. cerevisiae* strain used in the study of Chiaro *et al*. was poorly defined. The same concern applies to the strains/species used as controls. These consisted of *Rhodotorula aurantiaca* qualified as an “emerging pathogen”, but never reported as a pathogen to date, whereas C. *albicans* was, for undefined reasons, a 50:50 mixture of a wild strain and mutant for WOR1, a master regulator of white-opaque switching associated with *C. albicans* commensalism and pathogenesis[28].

Regarding the experimental model, although ASCA prompted the study, the presence of these antibodies was surprisingly not investigated in germ-free mice treated with DSS and producing UA following *S. cerevisiae* colonization by gavage. This contrasts with the results from at least two independent groups that demonstrated ASCA generation in mice colonized with *C. albicans* [29,30]. Indeed considering the relation between CD and anti-yeast mannan antibodies, it appears that according to the data accumulated over decades in basic and medical mycology *C. albicans* would be a more convincing suspect for CD triggering and/or maintenance than *S. cerevisiae*. In humans, we demonstrated that, through its well-known ability to modulate its oligomannose repertoire under pathogenic conditions, *C. albicans* was an immunogen for ASCA[31]. When investigating a large number of CD families we showed that CD and their first-degree healthy relatives (FDR) were more frequently colonized with *C. albicans* than control populations and in FDR the so far unexplained higher prevalence of ASCA with regard to control populations (30%) was associated with the carriage of *C. albicans* [31] In these studies, *C. albicans* represented 76–81% of the yeast species isolated compared to only 2–4% for *S. cerevisiae*. This increase in *Candida* abundance during CD in contrast with a reduction in *S. cerevisiae* revealed by culture methods was recently confirmed through NGS micro-mycobiome analysis by two independent groups [4,5]. Thus, the involvement of *S. cerevisiae* in CD is not supported by epidemiological, pathophysiological or immunological arguments, nor by the unconfirmed correlation between ASCA and UA. Concerning UA, its involvement in the pathophysiology of IBD is not supported by any data and neither is the hypothesis that an improvement of CD patients on allopurinol could result from “preventive yeast-induced UA build-up in the intestine”. The adjunctive role of allopurinol therapy in patients receiving thiopurines probably results from its role as a xanthine oxidase inhibitor shifting the metabolism of azathioprine or mercaptopurine from the inactive metabolite 6-methylmercaptopurine to the pharmacologically active metabolite, 6-thioguanine nucleotide [32].

In conclusion, we failed to confirm the clinical link between UA levels and antibodies against *S. cerevisiae* (ASCA) reported by Chiaro *et al*. [6], that this species negatively affected the course of CD. It is clear that much has still to be learnt about the involvement of the fungome in IBD and the complex metabolic interactions between yeasts and mammals.
